# The impact of a mixed reality technology-driven health enhancing physical activity program among community-dwelling older adults: a study protocol

**DOI:** 10.3389/fpubh.2024.1383407

**Published:** 2024-05-14

**Authors:** Michael Joseph S. Dino, Kenneth W. Dion, Peter M. Abadir, Chakra Budhathoki, Chien-Ming Huang, William V. Padula, Cheryl R. Dennison Himmelfarb, Patricia M. Davidson

**Affiliations:** ^1^School of Nursing, Johns Hopkins University, Baltimore, MD, United States; ^2^Research, Development, and Innovation Center, Our Lady of Fatima University, Valenzuela, Philippines; ^3^Sigma Theta Tau, International Honor Society in Nursing, Indianapolis, IN, United States; ^4^School of Medicine, Johns Hopkins University, Baltimore, MD, United States; ^5^Department of Computer Science, Johns Hopkins University, Baltimore, MD, United States; ^6^Department of Pharmaceutical and Health Economics, University of Southern California School of Pharmacy, Los Angeles, CA, United States; ^7^Office of the Vice Chancellor and President, University of Wollongong, Wollongong, NSW, Australia

**Keywords:** humanoid technologies, health-enhancing physical activity, healthy lifestyle, older adults, Fourth Industrial Revolution, mixed method research

## Abstract

**Background:**

Physical inactivity and a sedentary lifestyle among community-dwelling older adults poses a greater risk for progressive physical and cognitive decline. Mixed reality technology-driven health enhancing physical activities such as the use of virtual coaches provide an emerging and promising solution to support healthy lifestyle, but the impact has not been clearly understood.

**Methods and analysis:**

An observational explanatory sequential mixed-method research design was conceptualized to examine the potential impact of a user-preferred mixed reality technology-driven health enhancing physical activity program directed toward purposively selected community-dwelling older adults in two senior centers in the Philippines. Quantitative components of the study will be done through a discreet choice experiment and a quasi-experimental study. A total of 128, or 64 older adults in each center, will be recruited via posters at community senior centers who will undergo additional screening or health records review by a certified gerontologist to ensure safety and proper fit. Treatments (live coaching with video-based exercise and mixed reality technology-driven exercise) will be assigned to each of the two senior center sites for the quasi-experiment. The participants from the experimental group shall be involved in the discreet choice experiment, modeling, and usability evaluations. Finally, a qualitative sample of participants (*n* = 6) as key informants shall be obtained from the experimental group using purposive selection.

**Discussion:**

This study protocol will examine the health impact of a promising mixed reality program in health promotion among older adults. The study utilizes a human-centered mixed method research design in technology development and evaluation in the context of developing nations.

**Clinical trial registration**: ClinicalTrials.gov, identifier NCT06136468.

## Introduction

Physical inactivity, is common among older adults, affecting one-third of the global population (1, 2), and accounts for an estimated $53.8 billion global economic burden on the healthcare system (3, 4). It is also responsible for around 10% of global deaths annually (4). In the United States alone, almost 80% of older adults are considered inactive (5). Recent literature reviews reported a radical change in older adults’ lifestyles during the COVID-19 pandemic that further reduced physical activity (6, 7). In the Philippines, a survey reports that 67% percent of older adults are inactive, further exacerbated by the current pandemic (8, 9). The limitation on physical activity progresses physical deterioration and development of comorbidities and is considered a decisive risk factor for all-cause mortality (10). As an exemplar, the odds of COVID-19 death are 2.49 higher for inactive and sedentary patients (11). Therefore, there is a solid call to promote exercise programs that foster physical activity engagement and reduce sedentary lifestyle in older adults, most especially in the post-pandemic era (8, 12) since older adults are particularly susceptible of leading inactive lifestyles (13).

Forms of health-enhancing physical activity (HEPA) (14) like structured physical exercise are essential factors in maintaining normal body function, healthy aging, and promoting a longer life span (15). Regular exercise can help reduce frailty, fall risk, and premature morbidity. It is also relevant in preventing and managing health conditions common in the older adult population, such as hypertension, diabetes, obesity, insomnia, depression, and anxiety. Multinational campaigns and guidelines recommend the most beneficial multimodal exercise programs consisting of aerobic, resistance training, flexibility, and balance components (16). However, despite the tremendous benefits of HEPA, such as physical exercise, studies show that older adults are not actively engaged in such heath activities. In addition to the lack of a structured and enticing program, scholars reported several socio-cognitive processes that impact exercise behavior, adoption, and maintenance (17). In support, scientists have long established the critical role of culture, society, and environment in the success of HEPA programs (18). This outcome reinforces the development of location and culture-specific country programs based on the cohort preferences and national policy to support older adults’ HEPA (13), which are currently few and still underdeveloped (19).

As healthcare institutes recommend HEPA initiatives as essential components of health promotion for older adults, tools, and technologies supporting active lifestyles are increasing in parallel. Population aging and technological diffusion are two intersecting transitions currently experienced by the world. More than ever, the increased aging of the population requires technology-driven nursing services (20) to complement the demand for quality health services, especially for older adults with declining health and physical and mental challenges (21, 22). For instance, the introduction of Fourth Industrial Revolution technologies for health promotion for older people represents an emerging and promising intervention (23). Furthermore, numerous HEPA activities geared towards balance and muscle strength can be performed at home or on-site using technology tools such as health trackers and gadgets (24). In addition, multiple online resources that feature virtual guides are emerging and available.

Emerging electronic health (eHealth) interventions have centered on behavioral change relating to physical activity. They have shown effectiveness in many instances (25). The latest innovations in technology-driven HEPA are the utilization of humanoid (human-resembling) technologies (HTs) like physical humanoid robot coaches (HRCs) (22, 26). Due to the advent of virtual worlds and mixed reality applications, virtual digital assistants as embodied conversational agents are also emerging (25). Digital “coaches” in exercise projected through mixed reality displays are particularly useful in situations where social contact might be limited, such as during the pandemic through remote coaching (27). In addition, companies are now investing in developing technologies whose main structure is the fusion of the virtual and physical world in a “metaverse” (28). Health experts have arrived at a consensus that the emergence of metaverse applications will provide a promising future in delivering healthcare in all “spaces” and “dimensions” (29). The emergence of virtual coaches and metaverse studies in nursing is still novice but growing with increasing acknowledgment of opportunities and challenges surrounding its implementation (30–32).

While mixed reality technology-driven health enhancing physical activities has been previously shown to reduce the risk of diseases (33, 34), studies focusing on its impact on physical and mental functioning among older adults are still limited and scarce (35, 36). Scholars also reported a substantial divide among users and non-users of technologies for physical health (37). Furthermore, technology-lead interventions have been previously reported to be potentially susceptible to disregard due to usability issues and task quality. As a result, current studies are recommended to focus on the development and evaluation of user-preferred technologies via mixed-method inquiry (25, 38) to understand better how older adults interact with the technology and leverage the technology to meet their needs (22).

## Objectives

In response to the current gray spots and gaps found in a recently published literature review (31), this study envisions to advance the science of mixed reality technology-driven health interventions in the field of nursing by examining its impact through an exercise program directed toward community-dwelling older adults. Specifically, it aims to:

*Quantitative aim 1*: to assess older adults’ preferences for an ideal mixed-reality-driven virtual coach program based on several attributes and levels. The subjects are hypothesized to select age and ability-specific choices based on previous exposure to technology and life experiences.
*Quantitative aim 2*: to evaluate the impact of the mixed-reality-driven virtual coach program on participants’ health. The causal relationship between the outcome variables will be obtained from valid and reliable tools. Older adults with active participation in the program groups are hypothesized to elicit comparable physical and cognitive performances.
*Quantitative aim 3*: to model the older adults’ perception of the mixed-reality-driven virtual coach program usability and predictors of intention to participate in the program. The subjects are postulated to show indications of higher behavioral intention to participate in the program as affected by various variables.
*Qualitative aim 1*: to understand the older adult participants’ experiences during the mixed-reality-driven virtual coach program. A narrative descriptive qualitative approach will be carried out to evaluate the challenges or difficulties and successes or benefits of the program among those who benefitted the least and most from the intervention. The older adult key informants are expected to story-tell their practices focusing on themes related to acceptability, barriers, and facilitators.

Overall, tt was hypothesized that: (a) the older adult participants will identify their technology preferences based on their previous experiences and combination of various attributes, and (b) the use of mixed reality technology in physical exercise program will produce comparable health assessments when compared with the traditional form of health enhancing physical activities.

Human-computer interaction (HCI) is core to ensuring the usability of technologies and their successful integration to practice (39–42). Previous literature (43, 44) highlighted that HCI directly impacts technology users’ behavioral intention and actual utilization (44). Therefore, the adapted HCI model (45) offers a robust framework for the study. This model highlights a multitude of essential factors that needs to be considered in technology adoption such as content (user-preference), computer (technology features) and context (individual experiences and client uniqueness) that serves as antecedents of successful technology use in healthcare.

## Methods

### Design

An explanatory sequential mixed-method research design will be used to understand the impact of the mixed-reality-driven virtual coach program community-dwelling Filipino older adults and compare its effects against the current exercise program (live coaching and video-based) on the participants’ health. The current study advances four (4) research phases that will make use of distinct research approaches. Phases 1–3 shall adopt a post-positivist approach (46) through discreet choice experiment (conjoint analysis), quasi experiment, and structural equation modeling. Phase 4 follows a qualitative-constructivist paradigm (47) using descriptive qualitative research.

#### Phase 1: older adults’ preference for an ideal mixed-reality-driven virtual coach program

##### Discrete choice experiment

Understanding how older adults value the essential components of healthcare interventions through the conjoint method is crucial to both the design and evaluation of the program (48). Discrete choice experiment (DCE) or conjoint analysis involves measuring psychological judgment between choices and alternatives. It is also powerful in understanding and predicting the technology users’ attribute tradeoffs, decisions, and preferences of technology features (49). DCE was initially introduced in psychology and marketing but has gained popularity in medicine and healthcare (50). The adaptive conjoint analysis method is the most appropriate for the current study due to its practical and respondent-friendly features (51). This study will follow the published standards in applying the conjoint analysis methods in healthcare (52). In addition, it will include essential steps in the development of the adaptive conjoint survey, as follows: (a) defining HT-HEPA attributes and levels based on literature evidence, and (b) designing the attributes and levels as components of the HT-HEPA program, and (c) designing the conjoint survey questionnaire. The computer-based survey questionnaire using the Sawtooth software will be distributed to participants.

#### Phase 2: impact of mixed-reality-driven virtual coach program on participants’ physical, cognitive status, and QoL

##### Quasi experiment

Quasi experimental research will be employed to estimate the effect and examine the causal relationship between the HT-HEPA intervention and the study respondents’ physical, cognitive status, and QoL. Quasi experiments are appropriate for practical situations and ethical compliance for studies involving vulnerable participants such as older adults (53), and allow scholars to conduct rigorous studies under certain limitations and non-control conditions (54).

#### Phase 3: mixed-reality-driven virtual coach program’s system usability and predictors of intention to program participation

##### Structural equation modeling (SEM)

The popularity of structural equation modeling (SEM) as a research approach is attributed to its capacity to concurrently evaluate the validity of the measurement (55) while testing the relationship between latent variables (56). Specifically, the variance-based partial least squares (PLS-SEM) technique shall be used in the current study to maximize statistical power and deliver better convergence behavior (57, 58). UTAUT, usability, and behavioral intention variables will be processed to create a parsimonious model. An independent usability testing (59) with descriptive analysis will also be carried out.

#### Phase 4: participants’ experiences: program acceptability, barriers, and facilitators

##### Descriptive qualitative

Descriptive qualitative research is mainstream in studies exploring human phenomena in nursing and healthcare practice (60). In capturing the participants’ lived experiences with the technology, a descriptive qualitative tradition with thematic analysis (61) shall be applied. A descriptive approach, based on Husserlian principles (62), is proven powerful in clarifying poorly understood concepts of experiences (63), such as the potential acceptance, barriers, and facilitators of mixed reality health enhancing physical activity participation.

### Study setting, subject, and eligibility criteria

The study will be conducted at two senior centers in urban settings in the Philippines. The senior centers are served by a university-based medical facility located in the northern (Valenzuela City) and southern part (Quezon City) of Luzon Island in the Philippines, where the highest densities of older adults are found.

**Figure 1:**
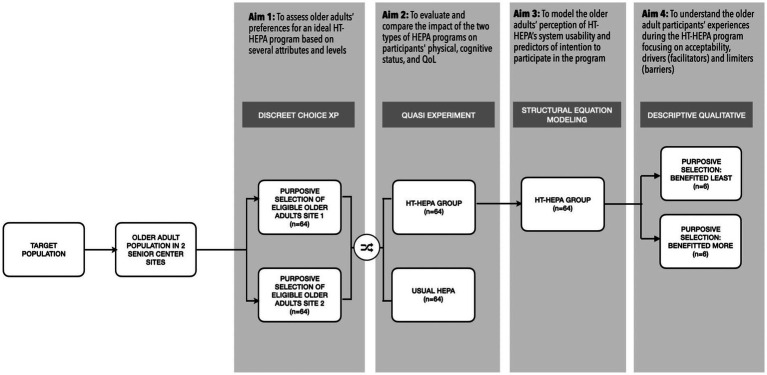
Study sampling plan.

The eligible participants will be purposively selected from the regular attendees at the study sites using the inclusion criteria: (1) ambulatory, (2) can follow simple instructions, (3) normal eyes functioning without low vision (or at most with corrective lenses), and (4) willingness to participate in the program with signed informed consent. Older adults ages 60–75 regardless of the gender and who are considered fit to participate by the community physician will be targeted. A total of 128 or 64 older adults in each center, will be recruited via posters at community senior centers who will undergo additional screening or health records review by a certified gerontologist to ensure safety and proper fit. Afterward, the treatments will be assigned to each of the two senior center sites for the quasi-experimental study. Only the participants from the experimental group shall be involved in the usability evaluations and qualitative arm. The sampling plan is shown in Figure 1.

### Informed consent and client involvement

Clients will be signing the printed informed consent and will be involved in the design and conduct of the study. An orientation will be conducted to communicate the research objectives, outcome measures, recruitment methods, and other relevant information. Client experience and preferences will be identified and incorporated.

## Interventions

**Figure 2:**
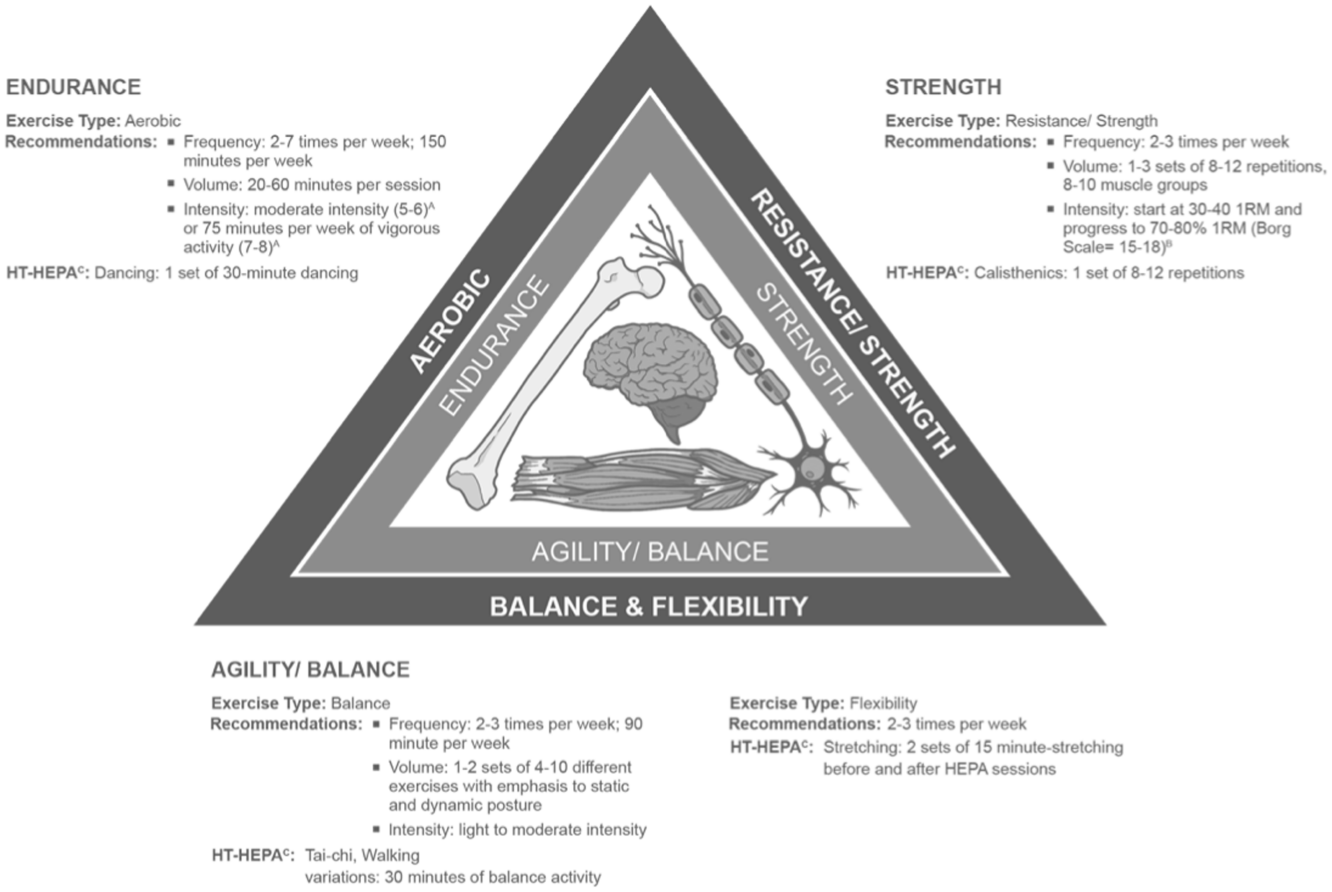
Summary of best practice recommendations for HEPA program for older adults.

**Figure 3:**
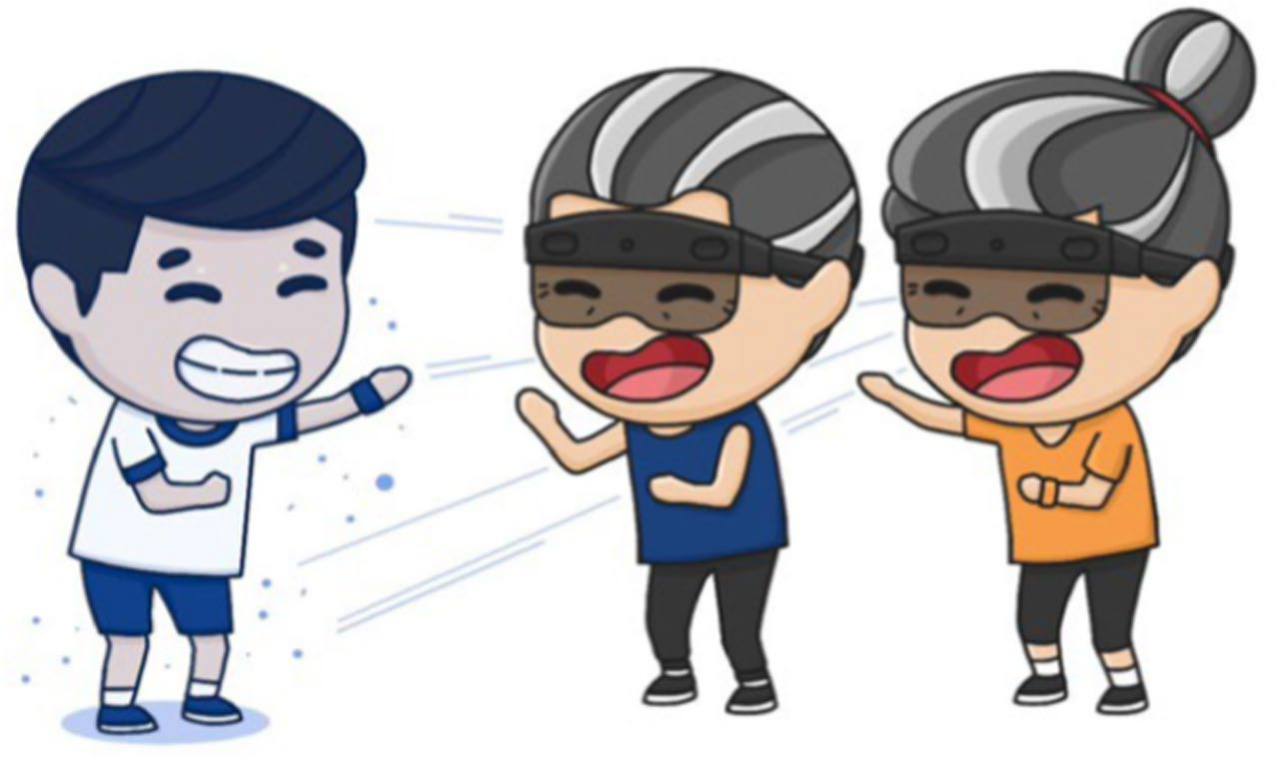
Concept diagram of virtual coach projected via see-through head-mounted-display.

This study will involve the development of mixed reality technology-driven virtual coach exercise program. The program shall be called “Hataw at Sigla para kay Lolo and Lola” (Groove and Move for older Adults), and will consist of the following procedures:

*Identifying the standard exercise for Filipino older adults*: the Philippines, similar to other territories, recommend a multimodal exercise as the most ideal program to support and improve the health of older adults (15, 64). This multimodal approach consists of aerobic, resistance, balance, and flexibility maneuvers (16). Developing the exercise program considers the uniqueness of the older adult cohorts with specific needs and requirements and compliance with recommended well-balanced exercise regimens (16). Results of systematic reviews (65, 66) and international expert consensus (64) shall serve as references for the age-appropriate program. The summary of the exercise components and recommendations are presented (Table 1; Figure 2). The ideal duration of the program is 1 h with 15 min of warming up and stretching before and after the session. This session length concurs with the current local recommendations (67, 68).
*HT programming*: this study will use a virtual humanoid “digital coach” to be projected using an optical see-through head-mounted-display (OST-HMD), Microsoft^®^ Hololens (see Figure 3). The device has been the most dominant, popular, and fastest developing mixed reality display in healthcare research since its release in 2016 (52, 69) due to its commendable inbuilt processing units and more extensive network of developers in healthcare (70). The device has been used for health and education programs for older adults in previous studies. The Mixed Reality Toolkit version 2, Unity 2018.4.x, and Unreal Engine are the primary software to be used.

**Table 1:** Summary of best practice recommendations for HEPA program for older adults.

Physical function	Exercise type	Recommendations	HT-HEPA^c^
Strength	Resistance/ Strength	Frequency: 2–3 times per week Volume: 1–3 sets of 8–12 repetitions, 8–10 muscle groups Intensity: start at 30–40 1RM and progress to 70–80% 1RM (Borg Scale = 15–18)^b^	Calisthenics: 1 set of 8–12 repetitions
Agility/ Balance	Balance	Frequency: 2–3 times per week; 90 min per week Volume: 1–2 sets of 4–10 different exercises with emphasis on static and dynamic posture Intensity: light to a moderate intensity as tolerated	Tai-chi, Walking exercise variations: 30 min of balance activity
	Flexibility	2–3 times per week	Stretching: Two sets of 15 min-stretching before and after HEPA sessions
Endurance	Aerobic	Frequency: 2–7 times per week; 150 min per week Volume: 20–60 min per session Intensity: moderate intensity (5-6)^a^ or 75 min per week of vigorous activity (7-8)^a^	Dancing: 1 set of 30-min dancing

a Scale of 0–10, where 0 is the level of effort for sitting, 10 is maximal effort, 5–6 is moderate, and 7–8 is vigorous.

b Original borg scale of perceived exertion from 6 (easy) to 20 (maximal).

c Two sessions per week scheduled every Tuesday and Thursday for the first 2 weeks, and three sessions per week scheduled every Monday, Wednesday, and Friday on the second 2 weeks.

### Improving client adherence and post-trial care

The participants (experimental and control) will receive an honorarium for their participation in the study. Other benefits include on-site snacks, free consultation from healthcare providers, and access to wellness facilities where the intervention will be conducted. In addition, participants who will be completing the whole program will receive wellness and exercise program paraphernalia.

### Outcomes

The predictor, outcome, and related variables will be assessed using appropriate, valid, and reliable measurement tools as shown in Table 2.

**Table 2:** Tools, instruments, and measurements.

Variables/ Measures	Materials and instruments	Properties
Intervention (X)		
	Virtual HT: Hololens^®^ 2 (Microsoft Corp.); Mixed reality devices	Most promising optical see-through mounted display due to its technical possibilities for tracking, interaction, and display (71)
	Adaptive conjoint survey	Practical and respondent-friendly (51)
System usability	Post study system usability questionnaire	Reliable (0.94), valid, adaptable, and practical (72)
Technology acceptance	Universal theory of acceptance and use of technology (UTAUT) questionnaire	Construct ICRS greater than 0.70, valid and reliable (73)
Outcome (Y)		
Physical		
Physical status	6-min walk test	Interclass reliability ICC = 0.94 (74)
Cognitive		
Cognitive status	Montreal cognitive assessment (MoCA)	Test–retest reliability between 7.1 and 7.2 (ICC: 0.64), and excellent between 7.1 and 7.3 (ICC: 0.82) (75)
Quality of life		
HRQoL	SF-8 quality of life (QoL)	TRR = 0.73, 0.74; α = 0.92 (76, 77)
Others (Z)		
Demographics, literacy	Residence, gender, age, marital status, education, technology literacy	Socio-demographic determinants linked to promoting health (78)
Health behavior	Patient action inventory for self-care	α = 0.95, 0.97, 0.97 (79)
Geriatric depression	Geriatric depression scale	IC (α = 0.92); SE (0.92); SP (0.91); PPV (76%); NPV (0.97) (80)
Anxiety symptoms	Spielberger state–trait inventory (STAI)	ICC =0.82–0.95 (81)
Sleep quality	Pittsburg sleep quality index	SE = 0.90; SP = 0.87 (82)

### Data collection and timeline

This study is composed of three data collection steps:

*Step 1: preparatory*: this phase includes the necessary preparation to enrich the effectiveness and efficiency of data collection through (1) an initial visit to the senior center sites to establish a partnership with the administration, orientation, acquire permission, and establish rapport among the prospective subjects, (2) technical set-up and training, and (3) health screening and actual sampling.
*Step 2: quantitative evaluation of preferences*: this phase involves the initial assessment of the older adults’ preferences for the mixed reality technology-driven exercise program via conjoint analysis. Results will be generated to inform the mixed reality technology system programming for the system’s actual development and initial pilot. This will follow the baseline assessment of participant data, actual exposure to the intervention, and post-intervention assessment of the subjects. The intervention will run for a month consisting of the first 2 weeks of the twice-weekly (Tuesday and Thursday) schedule to be followed by the second 2 weeks of trice weekly (Monday, Wednesday, Friday) schedule. Each intervention session program will last approximately 1.5 h, including orientation, warm-up, and stretching. All participants will be supervised by the health team and trained personnel at the senior center and follow a standard protocol.
*Step 3: qualitative evaluation*: this phase focuses on the individual interviews to assess views and opinions of study participants. An *aide-memoire* will be constructed as an interview tool to capture the stories and experiences of the older adult participants. Specifically, a responsive interviewing approach (83) will be used. Individual interviews will be transcribed, coded, and thematized. Qualitative findings will be used to substantiate the quantitative outcomes of the study.

### Sample size

To identify the acceptable sample size, the difference in repeated measures between two independent groups using MANOVA with a power set at 0.80 with medium to high effect size at 0.05 alpha level was computed using R software. The estimated sample size is 128 or 64 participants per senior center group.

## Data collection, management and statistical methods

A robust research data management (84) and analysis plan will be implemented to maintain the privacy, confidentiality, integrity, validity, and reliability of the gathered data in all stages of the data lifecycle (84). Only members of the research group will be given access through a double authentication procedure. The institutions currently utilize the latest Sophos SG series UTM firewall system and the Sophos UTM 9 management system that provides comprehensive gateway protection, including network intrusion prevention management. Backup is done every 7 PM daily, and every server is connected to an uninterruptible power supply. In terms of data analysis, the following approaches shall be used:

### Assessment older adults’ preferences for an ideal mixed reality technology-driven health enhancing physical activity program

Descriptive statistics will be used to describe the respondent demographics. In the conjoint method, regression analysis is adopted to relate the probability of choosing several profiles simultaneously. The regression model assumes that “the probability of choosing one profile is a linear function of the attribute levels in the profile” (85). Sawtooth Software Lighthouse Studio version 9.11 statistical package will be employed to calculate individual preference coefficients (utilities) in each attribute level and attribute importance scores (79, 80). In addition, the primary and joint effects of the preference attributes, average importance, and utility values will also be generated (86). In interpreting and reporting the conjoint outcomes, the ESTIMATE checklist (85) developed by the ISPOR Conjoint Analysis Good Research Practices Task Force shall be used.

### Comparing the effects of the mixed reality technology-driven health enhancing physical activities with the usual health enhancing physical activities (live coach and video-based) on participants’ physical, cognitive status, and quality of life measures

#### Repeated measures analysis of variance (RM-ANOVA)

Repeated Measures Analysis of Variance (RM-ANOVA) will be used to investigate the effects of the type of intervention on health variables across three assessment points using SPSS version 22. Mauchly’s Test will be utilized to verify the sphericity assumption with degrees of freedom correction using the Greenhouse–Geisser method. Effect sizes will also be reported.

### Assessing the older adults’ perception of mixed reality technology-driven health enhancing physical activity system’s usability and predictors of intention to participate in the program

#### Structural equation modeling

Structural Equation Modeling (SEM) through Partial Least Squares (PLS) will be employed to model the older adults’ perception of HT-HEPA’s system usability and predictors of intention to participate in the program. PLS-SEM is the most appropriate considering the number of older adult participants because of its power to precisely examine and estimate relationships among a set of variables (87, 88). This study will adopt the published guidelines for analyzing, interpreting, and reporting SEM outcomes (89) using the PLS Graph software package (90). For PLS-SEM, the measurement model’s evaluation is based on the reliability, construct, and discriminant validity of the measures associated with individual variables (89). Construct reliability assessment via composite reliability and Cronbach’s alpha (91) will measure the extent to which a set of items is consistent in what it intends to measure (90, 92). Convergent validity, on the other hand, is a measure of measurement instrument quality that can be assessed using two approaches: (a) evaluating the statistical significance (*p* < 0.05) of the item loadings associated with the construct and should be equal to or greater than 0.50 (90, 91) and (b) the use of average variance extracted (AVE) that quantifies the amount of variance that a construct captures from its items relative to the amount due to measurement error (93), greater than.50 (91, 94). The average variance extracted (AVE) can also be used to test if an instrument has discriminant validity, as evidenced by the AVE of each construct greater than its correlations with the other constructs (95).

### Understanding the older adult participants’ experiences during the mixed reality technology-driven health enhancing physical activities program

#### Thematic analysis

Interview texts will be transcribed, coded, and analyzed using MAXQDA^®^ Analytics Pro (VERBI Software, Germany). Member checking procedure (96) and reflective logs (97) will be adopted to ensure trustworthiness and reflexivity, respectively within the research team. Reflective notes or memos will be created to accumulate ideas, concepts, and connections with the generated quantitative data. Themes and/or subthemes shall be developed and reported using the COREQ guidelines (98). A code book will be created and maintained.

### Dissemination plans

The outcomes and changes in the study will be shared with the clients and stakeholders through post-study result dissemination and newsletter at the senior centers.

## Discussion

This protocol paper outlines the objectives, rationale, methods, and design for a multiple phase study that will examine the impact of a mixed reality-driven intervention through an exercise program directed toward community-dwelling older adults.

There has been increased research on older adult representations and studies focusing on technology interventions in the past decades (99). Although this upward trend is considered favorable in general, concerns related to ethical issues and the welfare of older adults participants are on the rise (100, 101), more so in aging studies involving technology interventions (42, 102). Older adults are essential to study subjects and clients in healthcare but are considered a vulnerable cohort in research studies.

As with other studies involving technologies, potential risks include loss of autonomy, privacy, data protection, safety, isolation prevention, and user overstressing. To minimize the risks, proper orientation will be held. The research team will undergo training and acquire good clinical practice certificate. Procedures will align with the currently accepted standards by the Philippine Department of Health, the research team will recruit social workers and healthcare providers to monitor the interventions and for potential health and wellness issues during the study. Reporting untoward events will comply with the current protocol and standards of the Philippine Health Research Ethics Board. Also, measurement error can be a potential challenge to the study and will be addressed by frequent recalibration and testing of instruments. Internet service interruptions needed to load exercise modules from the system storage might be an issue. This will be addressed by downloading the programs to the local server at the senior center sites.

This study protocol will provide an outstanding reference for future researchers for potential replication in other settings, timeline, and territories. It may also offer an outline of the necessary steps to ensure a robust but practical approach in technology studies in healthcare. The protocol supports the value of interdisciplinary and cross-disciplinary efforts and partnership in advancing the knowledge in the field of technology, health, and informatics. Outputs informing policies and standards are expected to be generated in this protocol for practice and research methods improvement.

## Ethics statement

The studies involving humans were approved by Johns Hopkins Medicine Institutional Research Board. The studies were conducted in accordance with the local legislation and institutional requirements. Written informed consent for participation in this study was provided by the participants. Written informed consent was obtained from the individual(s) for the publication of any potentially identifiable images or data included in this article.

## Author contributions

MD: Writing – original draft, Writing – review & editing. KD: Writing – original draft, Writing – review & editing. PA: Writing – original draft, Writing – review & editing. CB: Writing – original draft, Writing – review & editing. C-MH: Writing – original draft, Writing – review & editing. WP: Writing – original draft, Writing – review & editing. CH: Writing – original draft, Writing – review & editing. PD: Writing – original draft, Writing – review & editing.
